# BMI and Bioelectrical Impedance Analysis: Body Composition Assessment Identifying Elevated Body Fat in Normal-Weight Young Adults

**DOI:** 10.3390/nu18071060

**Published:** 2026-03-26

**Authors:** Róbert László Nagy, Bence Bombera, Viktor Rekenyi, Csongor István Szepesi, Nóra Horváth, Zsófi Balogh, László Róbert Kolozsvári

**Affiliations:** 1 Faculty of Medicine, University of Debrecen, 4032 Debrecen, Hungary; nagy.robert@hook.hu (R.L.N.); baloghzsofi0223@gmail.com (Z.B.); 2 Department of Family and Occupational Medicine, Faculty of Medicine, University of Debrecen, 4032 Debrecen, Hungaryrekenyi.viktor@med.unideb.hu (V.R.); szepesi.csongor@med.unideb.hu (C.I.S.); horvath.nora@med.unideb.hu (N.H.)

**Keywords:** dietary habits, body composition, bioelectrical impedance analysis, food frequency questionnaire, young adults, obesity, skeletal muscle mass, visceral fat, nutrition assessment, physical activity, IPAQ

## Abstract

Background: Body mass index (BMI) is commonly used to assess nutritional status; however, it cannot distinguish between fat and lean tissue. In young adults, this limitation may mask excess adiposity and distort diet–adiposity associations. Bioelectrical impedance analysis (BIA) provides more detailed measures, including percent of body fat (PBF), skeletal muscle mass (SMM), and the visceral fat level. Objectives: To examine how combining BMI with BIA-based classifications of adiposity influences the assessment of diet–body composition associations in young adults. Methods: This cross-sectional study of 285 young adults (median age 18 years, IQR: 18–20) used InBody BIA to classify participants by BMI and PBF. Dietary habits were assessed via food frequency questionnaire covering eight food groups. Group comparisons used Mann–Whitney U tests with Cohen’s d effect sizes; correlations used Spearman’s rank correlation. Results: Thirty-five participants (12.3%) were BMI-Normal but PBF-High (normal BMI with elevated body fat), a phenotype missed by BMI screening; overall BMI-PBF agreement was 75.4%. Physical activity (IPAQ) correlated significantly with body composition markers, PBF (rho = −0.177, *p* = 0.003) and SMM (rho = +0.186, *p* = 0.002), but not with BMI (rho = +0.060, *p* = 0.310). BMI showed an inverse association with self-reported sweets consumption (rho = −0.138, *p* = 0.020), likely reflecting a reporting bias rather than true intake, as this pattern disappeared when examining actual adiposity (PBF: rho = +0.032, *p* = 0.591). Conclusions: Combining BIA with BMI may improve the detection of elevated body fat (12.3% prevalence of normal BMI with elevated body fat); BMI-based screening may not identify all individuals with elevated body fat. Physical activity associations support the complementary value of BIA alongside BMI. Apparent diet–BMI associations may be confounded by adiposity misclassification and reporting bias.

## 1. Introduction

The global prevalence of overweight and obesity has reached epidemic proportions, with the World Health Organization (WHO) reporting that worldwide obesity has nearly tripled since 1975; in 2022, an estimated 890 million adults were living with obesity, representing approximately 1 in 8 people globally [1]. Young adults, particularly university students, represent a critical demographic for obesity prevention, as dietary habits established during this life stage often persist into later adulthood [2]. The transition to university life is frequently accompanied by changes in eating behaviors, including increased consumption of convenience foods, irregular meal patterns, and reduced intake of fruits and vegetables [3].

While the relationship between overall dietary intake and body weight has been extensively studied, the associations between the consumption frequency of specific food groups and detailed body composition parameters remain less clear [4]. Traditional anthropometric measures such as body mass index (BMI) provide limited information about the distribution of fat and lean tissue, which have distinct metabolic implications [5]. Bioelectrical impedance analysis (BIA) offers a non-invasive, practical method for assessing multiple body composition compartments, including fat-free mass (FFM), skeletal muscle mass (SMM), visceral fat level (VFL), and total body water (TBW) [6]. Recent comparative studies have demonstrated that BMI and percent of body fat (PBF) classifications may yield different obesity prevalence estimates and identify different at-risk populations [7].

Bioelectrical impedance analysis (BIA) offers a practical alternative to BMI for assessing body composition. BIA measures the impedance of body tissues to a small alternating electrical current; because fat tissue has a higher impedance than lean tissue (which contains more water and electrolytes), BIA can estimate fat mass, fat-free mass, and total body water. Modern multi-frequency segmental BIA devices analyze impedance at multiple frequencies across different body segments, providing detailed compartmental analysis that single anthropometric indices cannot offer [5,6].

Central European populations, including Hungarians, face unique nutritional challenges characterized by a high consumption of processed meats, refined carbohydrates, and saturated fats, combined with a relatively low intake of fish and fresh vegetables [8]. Understanding how specific dietary components relate to body composition in this population could inform targeted public health interventions. Previous studies in this region have focused primarily on cardiovascular risk factors and metabolic syndrome, with limited attention paid to detailed body composition assessment using modern BIA technology [9].

### Research Questions

Based on the existing literature and the known metabolic effects of different macronutrients, we formulated the following research questions:

Research Question 1: Does BMI-based categorization demonstrate inverse associations between energy-dense food consumption and adiposity in young adults, suggesting fundamental limitations in using BMI for nutritional assessment?

Research Question 2: Do body composition measures derived from bioelectrical impedance analysis (percent of body fat, visceral fat level, and skeletal muscle mass) show biologically plausible positive associations between energy-dense food consumption and adiposity that BMI alone may not capture?

Research Question 3: Do individuals misclassified as healthy by BMI (normal BMI but elevated body fat) display food group consumption patterns that differ from truly healthy individuals, demonstrating the clinical relevance of body composition analysis in nutritional epidemiology?

The primary objective of this study was to compare BMI-based and BIA-based (PBF, VFL) categorization approaches for assessing diet–adiposity relationships in young adults. Secondary objectives included quantifying the discordance between BMI and PBF classifications and characterizing the food group consumption patterns of individuals misclassified by BMI.

## 2. Materials and Methods

### 2.1. Study Design and Participants

This cross-sectional study was conducted among university students in Hungary between October 2025 and December 2025. Participants were recruited through university health screening programs using convenience sampling. Inclusion criteria were (1) age 18–25 years, (2) enrolled as a university student, and (3) ability to provide informed consent. Exclusion criteria included (1) pregnancy or lactation, (2) implanted electronic devices (pacemakers), (3) diagnosed eating disorders, and (4) conditions affecting body composition measurement (e.g., lymphedema, amputation).

The final sample comprised 285 participants (121 males, 164 females) with a median age of 18 years (IQR: 18–20). Only participants with complete InBody body composition data were included. Sample size was determined by the availability of participants during the screening period. Post hoc power analysis indicated 99% power to detect correlation coefficients of |rho| ≥ 0.15 at α = 0.05.

### 2.2. Dietary Assessment

Dietary habits were assessed using a food frequency questionnaire (FFQ) capturing habitual consumption over the preceding three months. Participants rated their consumption frequency for eight food groups on a 7-point Likert scale: 0 = never, 1 = less than once per week, 2 = 1–2 times per week, 3 = 2–3 times per week, 4 = 3–5 times per week, 5 = 5–6 times per week, and 6 = daily or more. The assessed food groups were (1) poultry, (2) fish, (3) cheese and curd, (4) milk and dairy products, (5) eggs, (6) raw fruits and vegetables, (7) white bread and pastries, and (8) sweets, cakes, and snacks. The questionnaire was adapted from the Hungarian National Healthcare Guidelines for Clinical Dietetic Practice, ensuring alignment with nationally recognized food group categories [10]. These items were selected based on their relevance to Hungarian dietary habits and their potential associations with body composition [11].

The sweets and snacks category included sweet baked goods (cakes, pastries), chocolate, confectionery, biscuits, and sweet desserts. Savory snacks (chips, crackers, and salted nuts) were not included in this category. The FFQ was adapted from Hungarian National Healthcare Guidelines [10] and assessed eight predefined food groups. Red meat, nuts/seeds, legumes, and fats/oils were not assessed as separate categories, which represents a limitation of this instrument.

### 2.3. Body Composition Assessment

Body composition was measured using a direct segmental multi-frequency bioelectrical impedance analyzer (InBody 270S, InBody Co., Ltd., Seoul, Republic of Korea). The InBody 270S measures impedance at two frequencies (20 kHz and 100 kHz) across five body segments (right arm, left arm, trunk, right leg, and left leg). Unlike single-frequency devices, multi-frequency BIA distinguishes between intracellular and extracellular water, improving accuracy of fat-free mass estimation. The InBody 270S has been validated against dual-energy X-ray absorptiometry (DXA) in young adult populations, showing strong correlations for percent of body fat (r = 0.83–0.93) and fat-free mass (r = 0.95–0.98) [12,13]. To properly contextualize the validity of the BIA-derived estimates, we reviewed published Bland–Altman agreement data comparing InBody multi-frequency devices with DXA in adults. The literature reports a systematic negative mean bias for percent body fat (e.g., underestimation by approximately 3.1%), with wide 95% limits of agreement (e.g., spanning from +2.6% to −8.9%) [12]. Furthermore, significant proportional bias is often observed in these validation studies, indicating that the magnitude of error varies across different adiposity levels and body mass index categories [13]. While these published agreement data support the overall utility of BIA in the present study, the lack of direct paired DXA measurements in our specific sample precluded a direct Bland–Altman analysis, which is acknowledged as a limitation. Measurements were performed in the morning according to manufacturer guidelines, with participants in a fasted state (minimum 4 h), wearing light clothing, barefoot, and having removed all metal accessories.

Participants were measured in a standing position, with arms held slightly away from the torso at approximately 15° abduction and feet placed on the device’s electrode platforms, according to the manufacturer’s protocol. Contact electrodes were positioned on the thumbs and palms for the hands and the heels and balls of the feet for the lower extremities. Participants were instructed to remain still and avoid talking during the measurement.

The following parameters were recorded:

- Weight (kg) and height (cm);
- Body mass index (BMI, kg/m^2^);
- Percent of body fat (PBF, %);
- Body Fat Mass (BFM, kg);
- Fat-free mass (FFM, kg);
- Skeletal muscle mass (SMM, kg);
- Visceral fat level (VFL, level 1–20);
- Basal Metabolic Rate (BMR, kcal);
- Waist–Hip Ratio (WHR);
- Total body water (TBW, L);
- InBody Score (points).

BMI categories were defined according to WHO criteria: underweight (<18.5 kg/m^2^), normal weight (18.5–24.9 kg/m^2^), overweight (25.0–29.9 kg/m^2^), and obese (≥30.0 kg/m^2^) [1].

Elevated body fat was defined using sex-specific percent of body fat (PBF) cutoffs, ≥25% for males and ≥32% for females, consistent with established thresholds for increased health risk in young adults [14]. Participants were classified as having elevated body fat if their PBF exceeded these sex-specific thresholds regardless of BMI category. These sex-specific PBF thresholds were derived from the study by Romero-Corral et al. [14], which used dual-energy X-ray absorptiometry (DXA) as the reference method for body composition assessment in a large adult population sample.

### 2.4. Physical Activity Assessment

Physical activity was assessed using the International Physical Activity Questionnaire—Short Form (IPAQ-SF) [15]. Participants reported the frequency (days/week) and duration (minutes/day) of vigorous-intensity activities, moderate-intensity activities, and walking performed during the preceding 7 days. Sitting time was also recorded as hours per day.

Total physical activity was calculated as metabolic equivalent minutes per week (MET-min/week) using the following formula: MET-min/week = (Vigorous days × Vigorous minutes × 8.0) + (Moderate days × Moderate minutes × 4.0) + (Walking days × Walking minutes × 3.3). Participants were classified into three activity levels according to IPAQ scoring protocol: (1) HIGH activity (vigorous activity on at least 3 days, achieving at least 1500 MET-min/week, or 7 days of any combination, achieving at least 3000 MET-min/week); (2) MODERATE activity (3 or more days of vigorous activity, 5 or more days of moderate activity, or 5 or more days of walking); and (3) LOW activity (not meeting criteria for moderate or high categories).

### 2.5. Statistical Analysis

Descriptive statistics were calculated for all variables. Continuous variables were expressed as mean ± standard deviation (SD), and categorical variables as frequencies and percentages. Due to the ordinal nature of FFQ data and non-normal distribution of several body composition parameters (assessed by Shapiro–Wilk test), non-parametric statistical methods were employed.

Spearman’s rank correlation coefficient (rho) was used to assess bivariate associations between dietary variables and body composition parameters. Correlation strength was interpreted as weak (|rho| < 0.40), moderate (0.40–0.59), strong (0.60–0.79), or very strong (≥0.80) [16]. Given the exploratory nature of this study and the 88 correlations tested (8 dietary × 11 body composition variables), we report uncorrected *p*-values, but note that a Bonferroni-corrected significance threshold would be *p* < 0.000568 (0.05/88). Correlations surviving this correction are explicitly noted.

Multiple linear regression was performed to identify independent predictors of BMI, with age and all eight dietary variables entered as predictors. Model assumptions were verified, including linearity, homoscedasticity, and absence of multicollinearity. Variance inflation factor (VIF) values ranged from 1.07 (fish) to 2.34 (age), all below the conventional threshold of 5.0.

Group comparisons of dietary variables across BMI categories were performed using the Kruskal–Wallis H test; when significant, pairwise Mann–Whitney U tests with Bonferroni-adjusted alpha levels were conducted as post hoc analyses. Sex differences in dietary habits were assessed using the Mann–Whitney U test. Statistical significance was set at *p* < 0.05 (two-tailed). All analyses were performed using Python 3.12 [17], with scipy (v1.11), statsmodels (v0.14), pandas (v2.0), and matplotlib (v3.8) packages. Effect sizes for group comparisons were calculated using Cohen’s d, interpreted as small (0.20), medium (0.50), or large (0.80). Agreement between BMI and PBF classifications was assessed using chi-square test and Cohen’s kappa (κ). Binary logistic regression was performed to identify predictors of elevated body fat in normal-BMI individuals.

## 3. Results

### 3.1. Participant Characteristics

The demographic and anthropometric characteristics of the study sample are presented in Table 1. The sample included 121 males (42.5%) and 164 females (57.5%), with a median age of 18 years (IQR: 18–20). The mean BMI was 23.42 (SD 3.85) kg/m^2^, with the majority of participants (72.3%) classified as normal weight, 21.4% as overweight, 0.0% as underweight, and 6.3% as obese.

### 3.2. Dietary Habits

Table 2 presents the descriptive statistics for dietary frequency variables. Dairy products showed the highest mean consumption frequency (4.47 (SD 1.68), corresponding to approximately 3–5 times per week), followed by poultry (4.43 (SD 1.57)). Fish consumption was low (2.06 (SD 1.58)), consistent with known Central European food group consumption patterns.

### 3.3. Correlation Analysis

Spearman’s rank correlation analysis revealed 88 bivariate correlations between eight dietary variables and 11 body composition parameters. Twelve correlations reached statistical significance (*p* < 0.05), with two reaching high significance (*p* < 0.01). The significant correlations are presented in Table 3. One additional significant correlation was found between poultry consumption and the waist-to-hip ratio (rho = 0.119, *p* = 0.044). However, when applying Bonferroni correction for multiple comparisons (*p* < 0.000568), no correlations retained statistical significance, highlighting the exploratory nature of these findings.

**Figure 1:**
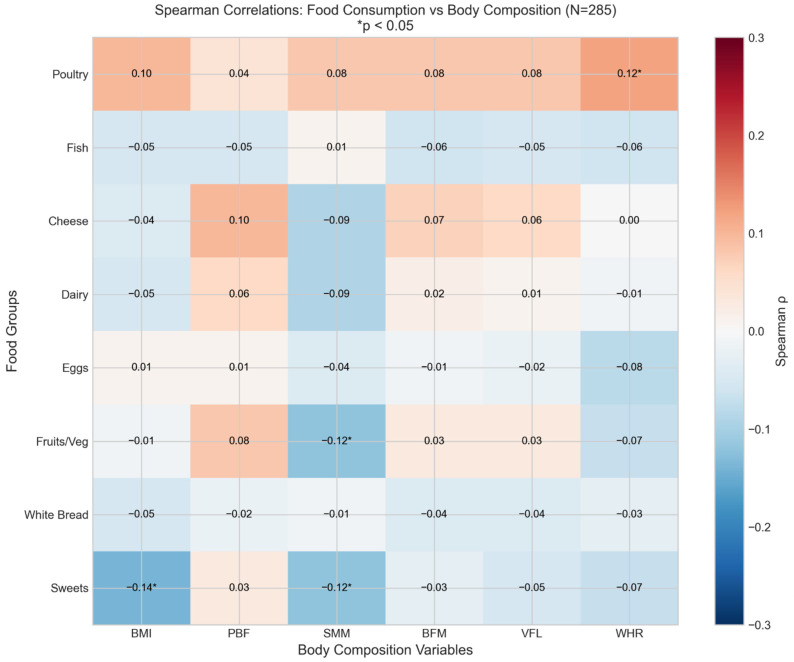
Correlation heatmap comparing dietary habits with BMI versus BIA-derived body composition parameters (PBF, VFL, and SMM). Note the opposite direction of sweets–BMI correlation (negative) versus sweets–PBF correlation (positive).

BMI-based analysis showed different patterns compared to PBF-based analysis (Figure 1): sweets and snacks consumption showed significant negative correlations with BMI (rho = −0.138, *p* = 0.020). In contrast, when examining the correlation with the percent of body fat (PBF), the association with PBF was positive but not statistically significant (rho = +0.032, *p* = 0.591). This divergence between BMI and PBF correlations illustrates the different information captured by these two measures of body composition (Figure 1, Table 4).

### 3.4. BMI vs. PBF Category Comparison

**Figure 2:**
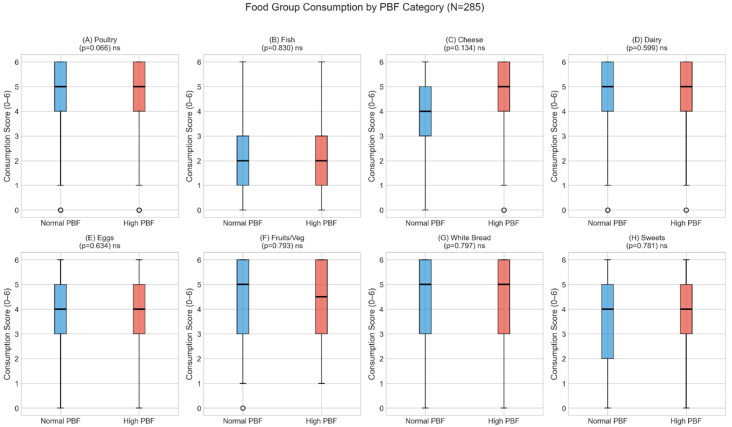
Box plots comparing dietary consumption by PBF category (normal vs. high). No significant differences in dietary consumption were observed between PBF categories.

To directly test our research questions, we compared food group consumption patterns between normal and high categories defined by both BMI and PBF (Table 5; Figure 2). Mann–Whitney U tests comparing food group consumption between normal and high categories revealed no significant differences for any of the eight food groups under either BMI-based (normal < 25 vs. high ≥ 25 kg/m^2^) or PBF-based (sex-specific cutoffs: male < 25%, female < 32%) classification (all *p* > 0.05; Table 5; Figure 2). Effect sizes were uniformly small to negligible (Cohen’s d range: 0.02–0.16 for BMI comparisons; 0.01–0.12 for PBF comparisons), indicating that neither classification approach detected meaningful differences in dietary consumption patterns between groups.

### 3.5. BMI-PBF Classification Agreement

Cross-tabulation revealed that BMI and PBF classifications agreed for only 75.4% of participants (Table 6). Of the 285 participants, 35 (12.3%) were classified as normal by BMI standards but high by PBF (normal weight with elevated body fat). BMI alone does not identify these individuals (Figure 2).

In line with our third research question, individuals misclassified by BMI (BMI-Normal but PBF-High) did not show significantly different food group consumption patterns compared to truly healthy individuals (both BMI-Normal and PBF-Normal).

### 3.6. Multiple Regression Analysis

Multiple linear regression was performed with BMI as the dependent variable and age, sex, plus all eight dietary variables as predictors (Table 7). The model explained 9.6% of the variance in BMI (R^2^ = 0.096, Adjusted R^2^ = 0.090; and F *p* < 0.001), meaning that demographic and dietary factors together account for approximately one-tenth of the variation in BMI values. For body fat percentage, the model explained 34.3% of the variance (R^2^ = 0.343, Adjusted R^2^ = 0.336), indicating that these same predictors explain roughly one-third of PBF variation—substantially more than for BMI. This higher explanatory power for PBF suggests that dietary factors may have a more direct relationship with actual body fat than with BMI.

Age and sex were significant independent predictors of BMI and body fat percentage. For each additional year of age, the BMI increased by 0.41 kg/m^2^ (β = 0.406, *p* = 0.002) and body fat percentage by 0.83% (β = 0.826, *p* = 0.005). Males had a significantly higher BMI (+1.891 kg/m^2^, *p* < 0.001) but lower body fat percentage (−11.4%, *p* < 0.001) compared to females. Fish consumption was not significantly associated with body fat percentage (β = −0.424, *p* = 0.169).

### 3.7. Food Group Consumption by BMI Category

Kruskal–Wallis tests comparing food group consumption across BMI categories (normal weight, overweight, and obese) revealed no significant differences for any food group (all *p* > 0.05). Specifically, neither dairy consumption (H = 3.14, *p* = 0.371) nor sweets/snacks consumption (H = 4.68, *p* = 0.197) differed significantly across BMI categories. This finding is consistent with the weak and largely non-significant bivariate correlations reported above.

### 3.8. Sex Differences

Mann–Whitney U tests revealed sex differences in raw fruits/vegetables consumption (males: 3.95 (SD 1.58), females: 4.49 (SD 1.65); *p* = 0.001). No significant sex difference was found for sweets/snacks consumption (males: 3.49 (SD 1.76), females: 3.79 (SD 1.69); *p* = 0.153). As expected, differences were found in body composition parameters related to body size and lean mass (Table 8).

### 3.9. Physical Activity and Body Composition

IPAQ-SF data revealed that participants had a mean MET-min/week of 3227 (SD 2362), with a median of 2779 MET-min/week. Activity level classification showed the following: LOW 10 (3.5%), MODERATE 133 (46.7%), and HIGH 142 (49.8%). Spearman’s rank correlation analysis revealed notable associations between physical activity (MET-min/week) and body composition measures (Table 9).

**Figure 3:**
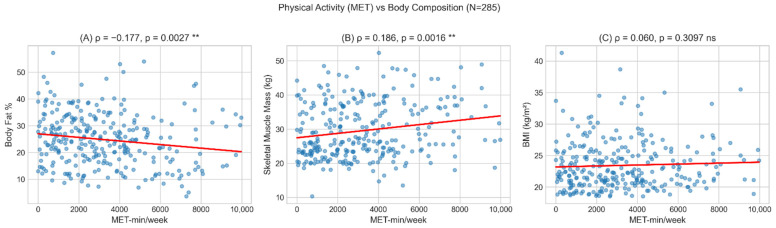
Scatter plots showing MET-min/week correlations with body composition measures. (**A**) MET vs. PBF shows significant negative correlation (rho = −0.177, *p* = 0.003). (**B**) MET vs. SMM shows significant positive correlation (rho = +0.186, *p* = 0.002). (**C**) MET vs. BMI shows no significant correlation (rho = +0.060, *p* = 0.310). The red solid line represents the linear regression line (line of best fit), illustrating the overall trend and direction of the association between physical activity and the respective body composition parameter ** *p* < 0.01; ns = not significant; the red line indicates the linear trend.

**Figure 4:**
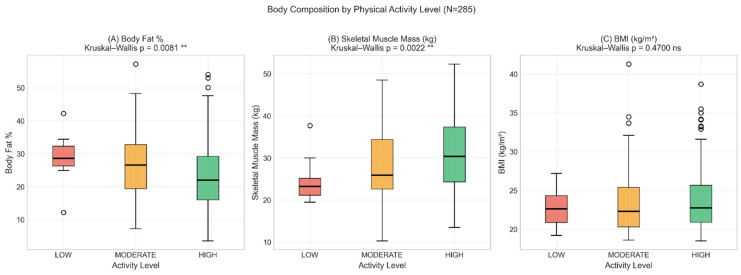
Body composition by activity level (LOW, MODERATE, and HIGH). (**A**) PBF decreases with higher activity (*p* = 0.008). (**B**) SMM increases with higher activity (*p* = 0.002). (**C**) BMI shows no significant difference (*p* = 0.470) ** *p* < 0.01; ns = not significant.

Physical activity correlated with PBF, SMM, and FFM (Figure 3), but there was no significant correlation with BMI (rho = +0.060, *p* = 0.310). This finding suggests that BMI alone does not capture body composition changes associated with physical activity, supporting the complementary use of BIA. Kruskal–Wallis tests comparing body composition by activity level confirmed this pattern: differences were found for PBF (*p* = 0.008) and SMM (*p* = 0.002) but not for BMI (*p* = 0.470) (Figure 4).

### 3.10. Exploratory Subgroup Analysis: High-BMI/Low-Sweets Phenotype

To explore whether the inverse BMI–sweets association could be explained by an athletic body composition, we identified a subgroup of 56 individuals with both a high BMI (≥25) and low sweets consumption (≤median). This exploratory analysis examined whether this subgroup represents muscular individuals misclassified by BMI. Compared to other participants, this subgroup had markedly higher skeletal muscle mass (35.7 vs. 27.7 kg, *p* < 0.001), suggesting that some high-BMI individuals are muscular rather than obese.

However, this subgroup did not show significantly different physical activity levels (MET 3297 vs. 3209, *p* = 0.826), suggesting that the body composition of these individuals may reflect constitutional factors rather than exercise-induced changes. The group with normal BMI but elevated body fat (BMI-Normal but PBF-High, N = 35) did not show significantly different physical activity levels (MET 2799 ± 2423 vs. 3224 ± 2293, *p* = 0.22).

### 3.11. Extended Regression: MET as Independent Predictor

When MET-min/week was added to the regression model for PBF (Table 7, final panel), the MET did not reach statistical significance as an independent predictor (β = −0.0003, *p* = 0.165), with the model explaining 34.7% of the variance (R^2^ = 0.347). The addition of physical activity did not meaningfully improve model fit beyond age and sex.

### 3.12. Logistic Regression: Predictors of Elevated Body Fat in Normal-BMI Individuals

To identify predictors of normal weight with elevated body fat (BMI-Normal but PBF-High), a binary logistic regression was performed among participants with a normal BMI (*n* = 206). The model included age, sex, physical activity (MET-min/week), and sweets consumption frequency as predictors (Table 10).

Sex was the only statistically significant predictor of a normal BMI with elevated body fat: females had significantly higher odds of being classified as having elevated body fat despite having a normal BMI compared to males (OR = 11.75, 95% CI: 2.71–50.90, and *p* = 0.001). Neither age, physical activity, nor sweets consumption frequency were significant predictors. Notably, sweets consumption showed no association with this phenotype (OR = 1.07, *p* = 0.598), consistent with the overall finding that self-reported dietary intake has limited predictive value for body composition in this population.

## 4. Discussion

This cross-sectional study examined the relationship between food group consumption and body composition in 285 young Hungarian adults, comparing BMI-based and BIA-based classification approaches. The study addresses a methodological gap in nutritional epidemiology, where BMI remains the predominant measure that does not distinguish between fat and lean tissue mass [5,6].

Methodological note on sweets/snacks as an indicator food group: Throughout this discussion, sweets and snacks consumption serves as an indicator variable to demonstrate how classification method (BMI vs. PBF) affects the direction of diet–adiposity associations. We do not claim that sweets consumption is a primary obesogenic factor in this population; rather, it provides a useful test case, because it is commonly assumed to associate positively with adiposity. The inverse BMI correlation illustrates the methodological limitation, regardless of any true causal relationship between sweets intake and body fat.

### 4.1. Reporting Bias and BMI Misclassification

The inverse association between self-reported sweets consumption and BMI (rho = −0.138, *p* = 0.020) does not reflect true dietary intake. Rather, this pattern demonstrates a well-documented phenomenon: individuals with a higher BMI tend to underreport consumption of socially undesirable foods (social desirability bias) [18,19]. This reporting bias, combined with BMI not distinguishing between fat and lean tissue [5,6,14], creates potentially misleading diet–BMI associations. Our data showed that the sweets–SMM correlation was also negative (−0.120), indicating that individuals with a lower lean body mass appear to consume more sweets in self-reports. Because BMI conflates muscle and fat [5,14], these measurement artifacts compound.

Evidence for the artifact: When examining actual adiposity (PBF) instead of BMI, this inverse pattern disappears (rho = +0.032, *p* = 0.591), suggesting that the BMI finding may be an artifact of measurement limitations and reporting bias rather than underlying biological associations. This direction reversal—from negative (BMI) to positive (PBF)—suggests that BMI-based dietary associations may be confounded by both adiposity misclassification [5,6,14] and differential reporting bias across BMI categories [18].

### 4.2. Physical Activity Supports BMI Limitation Evidence

The integration of IPAQ-SF physical activity data provides additional evidence for the limited sensitivity of BMI alone. Physical activity (MET-min/week) showed significant correlations with true body composition measures (PBF: rho = −0.177, *p* = 0.003; SMM: rho = +0.186, *p* = 0.002) but no significant correlation with BMI (rho = +0.060, *p* = 0.310). Individuals who exercise more have lower body fat and higher muscle mass, yet BMI alone may not capture these body composition differences.

The absence of an MET-BMI correlation can be explained by the compensatory effect: as exercise reduces fat mass but increases muscle mass, BMI remains relatively stable. This illustrates a limitation of the BMI as an adiposity measure: it does not distinguish between individuals who have achieved a given BMI through different pathways (high muscle/low fat vs. low muscle/high fat).

### 4.3. Normal Weight with Elevated Body Fat

Our BMI-PBF crosstab analysis revealed that 35 individuals (12.3%) were classified as normal by BMI but high by PBF. This phenotype, characterized by elevated body fat despite having a normal BMI, has been described in several populations [20,21].

Clinical implication: Using BMI alone for screening may not identify all individuals with elevated body fat. Combining BMI with BIA or other body composition assessment methods may increase screening sensitivity, particularly in young adult populations where the prevalence of elevated body fat with a normal BMI may be substantial.

### 4.4. Methodological Considerations

The sex-specific PBF cutoffs applied in this study (≥25% for males, ≥32% for females) were originally established using DXA [14], whereas body composition in the present study was assessed using multi-frequency BIA (InBody 270S). While DXA is considered a reference standard for body composition assessment, several validation studies have demonstrated acceptable agreement between the InBody 270S and DXA for estimating percent body fat and fat-free mass in healthy young adult populations, with reported correlations ranging from r = 0.92 to r = 0.98 for PBF [12,13]. Nevertheless, BIA estimates of body fat percentage may exhibit systematic bias relative to DXA, with the direction and magnitude depending on hydration status, ethnicity, and adiposity level. The InBody 270S tends to slightly underestimate PBF in lean individuals and overestimate it in those with higher adiposity compared to DXA [6]. Applying DXA-derived cutoffs to BIA-estimated PBF values may therefore introduce classification error, potentially affecting the prevalence estimates of elevated body fat with a normal BMI reported in this study. Future studies should ideally derive population-specific BIA cutoffs validated against DXA or apply device-specific correction equations to improve classification accuracy.

While our BMI-vs-BIA comparison provides evidence for BMI limitations, the cross-sectional design means reverse causality cannot be excluded—individuals with a higher body weight may actively restrict sweets consumption as a weight management strategy [22]. Self-reported dietary data are also susceptible to social desirability bias [18]; however, the divergent patterns between BMI and PBF (negative vs. positive correlations with sweets) suggest that BMI measurement limitations, rather than reporting bias, primarily explain our findings.

Statistical note on multiple comparisons: After Bonferroni correction, no individual correlations retained statistical significance. However, this study does not aim to establish specific diet–adiposity associations; rather, it demonstrates how BMI-based classification systematically distorts the directionality of associations even when effect sizes are small. The consistent pattern—negative correlations with BMI but positive (or neutral) correlations with PBF across multiple variables—supports our methodological conclusion regardless of individual *p*-values.

### 4.5. Comparison with Previous Studies

Our findings contrast with prospective studies demonstrating positive associations between sugar-sweetened beverage consumption and weight gain [22,23]. However, cross-sectional studies have occasionally reported similar findings. A study by Darmon and Drewnowski [24] found that obese individuals reported a lower consumption of sweets and fats compared to normal-weight participants, attributed to dietary underreporting and active restraint [25].

Sex differences in food group consumption patterns were observed, with females reporting a significantly higher intake of raw fruits/vegetables (*p* = 0.001).

### 4.6. Clinical and Public Health Implications

Despite the unexpected direction of some associations, our results have practical relevance. The complexity of diet–body composition relationships suggests that multiple assessment methods, including objective dietary biomarkers, may be needed. BIA-derived body composition parameters provide more detailed information than BMI alone, enabling differentiation between fat and lean tissue compartments.

The observed coefficients of determination (R^2^ = 0.096 for BMI, R^2^ = 0.343 for PBF) indicate that dietary factors explain a modest proportion of variance in body composition, consistent with the multifactorial nature of adiposity. Genetic predisposition, total energy intake, macronutrient distribution, sleep patterns, and psychosocial factors all contribute to body composition but were not assessed in this study. The modest R^2^ values should be interpreted in the context of using frequency-based dietary data rather than quantitative nutrient intake. Furthermore, regarding the bivariate associations shown in Figure 1 and Figure 3 and Table 9, the modest correlation coefficients (rho ranging from −0.177 to +0.186) translate to low individual coefficients of determination (r^2^ = 0.01–0.03). This highlights that no single behavioral metric explains a large portion of body composition variance but rather they act cumulatively.

The notable age effect on BMI (β = 0.406 per year) emphasizes the importance of early intervention, as even young adults show weight accumulation with increasing age. University health programs should consider targeted nutritional education that addresses both the quantity and quality of dietary intake.

Although our BIA device (InBody 270S) provided automated WHR estimates, simpler clinical anthropometric measures such as manual, tape measure-derived waist circumference (WC) and waist-to-height ratio (WHtR) may also identify individuals with elevated metabolic risk without the need for specialized equipment. These measures have demonstrated good predictive value for cardiometabolic outcomes in young adult populations and are recommended by several clinical guidelines. Future studies should explicitly compare the screening performance of traditional tape measure methods with BIA-derived parameters to determine the most cost-effective approach for population-level body composition assessment.

### 4.7. Strengths and Limitations

Several general limitations of BIA methodology for estimating body fat percentage warrant consideration. BIA derives body composition estimates indirectly from whole-body or segmental impedance measurements using proprietary prediction equations that are calibrated against reference populations [6]. The accuracy of these estimates depends on assumptions regarding the hydration of fat-free mass (typically assumed to be 73.2%), body geometry, and the proportional distribution of fluid between intracellular and extracellular compartments. Deviations from these assumptions—such as those occurring with acute changes in hydration status, recent physical exercise, food or fluid intake, menstrual cycle phase, or ambient temperature—can introduce measurement errors [6]. Furthermore, BIA prediction equations are population-specific; equations developed in one ethnic or age group may not generalize well to others, potentially introducing systematic bias when applied to Central European young adults. Multi-frequency segmental devices such as the InBody 270S partially mitigate some of these limitations by measuring impedance at multiple frequencies (distinguishing intracellular from extracellular water) and across individual body segments, thereby reducing reliance on geometric assumptions. However, the proprietary nature of InBody algorithms limits transparency regarding the reference populations and statistical models underpinning the output values. Additionally, BIA cannot distinguish between subcutaneous and visceral adipose tissue directly; visceral fat level estimates are derived from proprietary algorithms rather than direct measurement, which may reduce accuracy compared to imaging-based methods such as computed tomography or magnetic resonance imaging [5,6]. Despite these limitations, BIA remains a practical, non-invasive, and cost-effective tool for field-based body composition assessment, particularly in epidemiological studies involving large samples where DXA or imaging is not feasible.

Strengths of this study include the use of validated BIA technology for detailed body composition assessment, application of appropriate non-parametric statistical methods for ordinal dietary data, and a focus on the understudied young adult population. The inclusion of multiple body composition parameters beyond BMI provides a more complete picture of nutritional status.

Limitations must be acknowledged. First, the cross-sectional design precludes causal inference. Second, although individual dietary correlations were of small magnitude (Cohen’s d < 0.30), this is expected given the multifactorial nature of body composition and the limitations of self-reported dietary data. Third, self-reported dietary data are subject to substantial measurement error, including the systematic underreporting of energy-dense foods. Research consistently shows that individuals with higher adiposity tend to underreport consumption of socially undesirable foods such as sweets and snacks, a phenomenon known as social desirability bias [18]. This underreporting may explain why neither BMI-based nor PBF-based analyses revealed strong positive correlations between sweets consumption and adiposity measures. Fourth, convenience sampling from university health screenings may limit generalizability. Fifth, the FFQ assessed only eight food groups using a 7-point Likert scale, not capturing overall energy intake or portion sizes. Although the FFQ structure followed Hungarian National Healthcare Guidelines [10], it was not validated against dietary records or biomarkers, limiting our ability to quantify underreporting or assess true dietary intake. Sixth, we report uncorrected *p*-values for transparency. Given the exploratory nature and the high number of tests (88 correlations), we acknowledge that Bonferroni correction would eliminate all individual correlations. Moreover, the very low significance threshold after Bonferroni correction increases the risk of Type II errors, potentially rejecting genuinely existing associations. However, the primary findings (prevalence of normal BMI with elevated body fat = 12%, IPAQ–body composition associations) remain robust, as they do not rely on correlation thresholds. The dietary correlations serve to illustrate the limited sensitivity of BMI alone rather than establish causal diet–adiposity links. Future research should employ validated FFQs with energy intake estimation and consider prospective designs to establish temporal relationships between dietary habits and body composition changes.

Additionally, this study did not include clinical metabolic markers (fasting glucose, lipid panel, HOMA-IR, and blood pressure), which are essential for true metabolic phenotyping. Our classification of elevated body fat with a normal BMI is based solely on body composition parameters and does not constitute metabolic risk assessment. Future studies should combine BIA with metabolic biomarkers to determine whether the body composition discrepancies identified here correspond to actual cardiometabolic risk elevation.

## 5. Conclusions

This cross-sectional study demonstrates that BIA-based body composition assessment may provide additional value for detecting elevated body fat not identified by BMI alone. Physical activity (IPAQ) showed significant correlations with BIA-derived measures (PBF, SMM) but not with BMI, supporting the complementary value of BIA. The key finding is that 12% of participants had elevated body fat despite having a normal BMI, while apparent diet–BMI associations likely reflect reporting bias and adiposity misclassification rather than true nutritional relationships.

Four main conclusions emerge:

1. Elevated body fat in normal-weight individuals: 12.3% of participants had a normal BMI but elevated body fat percentage, a discrepancy that BMI alone did not detect. These individuals displayed body composition profiles that standard BMI-based assessment alone would not identify, supporting the complementary use of BIA in young adult populations.
2. Physical activity validates BIA: MET-min/week showed statistically significant correlations with PBF (rho = −0.177, *p* = 0.003) and SMM (rho = +0.186, *p* = 0.002) but no significant correlation with BMI (rho = +0.060, *p* = 0.310). This suggests that BIA captures body composition changes associated with physical activity that BMI alone does not reflect [26].
3. Dietary associations explained: The inverse BMI–sweets association (rho = −0.138, *p* = 0.020) may reflect reporting bias and BMI misclassification rather than underlying biological associations. When examining actual adiposity (PBF), this inverse pattern disappeared (rho = +0.032, *p* = 0.591), suggesting that BMI-based dietary associations are confounded by measurement artifacts.
4. Clinical implication: BIA may provide additional screening value [27] for identifying individuals with a normal BMI but elevated body fat (12.3% in this sample). Integrating BIA alongside BMI may improve the detection of body composition discrepancies.

These findings support integrating BIA alongside BMI in diet–adiposity research. Future studies should employ complementary body composition methods to more accurately assess relationships between food group consumption and obesity risk.

## Figures and Tables

**Table 1:** Participant characteristics (N = 285).

Variable	Mean (SD), Median (IQR), or *n* (%)	Range	Males (*n* = 121)	Females (*n* = 164)	*p*-Value
Demographics					
Age (years)	18 (IQR: 18–20) †	18–25	18 (IQR: 18–20) †	18 (IQR: 18–20) †	0.124
Sex (Male/Female)	121 (42.5%)/164 (57.5%)	N/A			
Anthropometric Measures					
Height (cm)	172.79 (SD 9.69)	150.0–198.0	181.56 (SD 6.52)	166.32 (SD 5.76)	<0.001
Weight (kg)	70.39 (SD 15.31)	41.6–135.0	80.82 (SD 13.77)	62.71 (SD 11.35)	<0.001
BMI (kg/m^2^)	23.42 (SD 3.85)	18.5–41.3	24.50 (SD 3.87)	22.63 (SD 3.66)	<0.001
Body Composition (BIA)					
Body Fat (%)	24.81 (SD 9.78)	3.6–57.2	18.38 (SD 7.99)	29.56 (SD 8.13)	<0.001
Body Fat Mass (kg)	17.44 (SD 8.37)	2.5–52.9	15.40 (SD 8.66)	18.95 (SD 7.85)	<0.001
Fat-Free Mass (kg)	52.96 (SD 13.39)	21.7–90.7	65.42 (SD 9.37)	43.76 (SD 6.89)	<0.001
Skeletal Muscle Mass (kg)	29.52 (SD 8.13)	10.3–52.3	37.14 (SD 5.64)	23.90 (SD 4.13)	<0.001
Visceral Fat Level	7.18 (SD 4.51)	1.0–24.0	6.09 (SD 4.32)	7.98 (SD 4.48)	<0.001
Basal Metabolic Rate (kcal)	1513.84 (SD 289.29)	840–2329	1783.13 (SD 202.23)	1315.15 (SD 148.84)	<0.001
Waist–Hip Ratio	0.87 (SD 0.07)	0.7–1.2	0.88 (SD 0.08)	0.86 (SD 0.06)	0.083
Total Body Water (L)	38.77 (SD 9.81)	16.0–66.2	47.93 (SD 6.82)	32.02 (SD 5.04)	<0.001
InBody Score (points)	74.85 (SD 8.97)	42–99	78.55 (SD 9.75)	72.76 (SD 7.70)	<0.001
BMI Categories					
Underweight (<18.5)	0 (0.0%)	N/A	0 (0.0%)	0 (0.0%)	<0.001
Normal (18.5–24.9)	206 (72.3%)	N/A	73 (60.3%)	133 (81.1%)	
Overweight (25.0–29.9)	61 (21.4%)	N/A	39 (32.2%)	22 (13.4%)	
Obese (≥30.0)	18 (6.3%)	N/A	9 (7.4%)	9 (5.5%)	

BMI, body mass index; BIA, bioelectrical impedance analysis; SD, standard deviation; and IQR, interquartile range. † Age reported as median (IQR) due to non-normal distribution (Shapiro–Wilk *p* < 0.001).

**Table 2:** Dietary frequency variables (0–6 scale).

Food Group	Mean (SD)	Median	Valid *n*
Dairy products	4.54 (SD 1.66)	5	285
Poultry	4.47 (SD 1.58)	5	285
Raw fruits and vegetables	4.26 (SD 1.64)	4	285
White bread, pastries	4.14 (SD 1.78)	5	285
Eggs	4.11 (SD 1.57)	4	285
Cheese and curd	4.17 (SD 1.62)	4	285
Sweets, cakes, snacks	3.66 (SD 1.72)	4	285
Fish	2.06 (SD 1.57)	2	285

Scale interpretation: 0 = never, 1 ≤ 1×/week, 2 = 1–2×/week, 3 = 2–3×/week, 4 = 3–5×/week, 5 = 5–6×/week, and 6 = daily.

**Table 3:** Spearman’s rank correlation matrix: diet–body composition associations.

Dietary Variable	Weight	TBW	FFM	BMR	SMM	BMI	Height	WHR	PBF	BFM	VFL
Poultry	+0.092	+0.066	+0.067	+0.067	+0.069	+0.099	+0.048	+0.127 *	+0.050	+0.092	+0.089
Fish	−0.019	+0.013	+0.011	+0.012	+0.011	−0.047	+0.020	−0.064	−0.053	−0.063	−0.057
Cheese and curd	−0.049	−0.090	−0.091	−0.091	−0.089	−0.037	−0.066	+0.002	+0.097	+0.070	+0.060
Dairy products	−0.067	−0.087	−0.088	−0.088	−0.087	−0.047	−0.093	+0.004	+0.064	+0.029	+0.019
Eggs	−0.043	−0.053	−0.054	−0.054	−0.050	+0.004	−0.084	−0.073	+0.016	+0.002	−0.014
Raw fruits/vegetables	−0.081	−0.118 *	−0.119 *	−0.119 *	−0.119 *	−0.005	−0.153 **	−0.060	+0.085	+0.037	+0.036
White bread, pastries	−0.018	+0.006	+0.004	+0.004	+0.005	−0.053	+0.034	−0.022	−0.039	−0.047	−0.050
Sweets/snacks	−0.153 **	−0.123 *	−0.123 *	−0.123 *	−0.120 *	−0.138 *	−0.090	−0.067	+0.032	−0.031	−0.050

Spearman rho values shown for all 8 food groups across 11 body composition parameters. PBF, Percent Body Fat; BFM, Body Fat Mass; VFL, visceral fat level; WHR, Waist-to-Hip Ratio; TBW, Total Body Water; FFM, Fat-Free Mass; BMR, Basal Metabolic Rate; and SMM, Skeletal Muscle Mass, BMI Body Mass Index. Significance markers: (*) *p* < 0.05; (**) *p* < 0.01.

**Table 4:** Sweets/snacks correlations: BMI vs. body composition measures.

Body Composition Measure	Spearman’s Rank Correlation	Direction	Interpretation
BMI	−0.138 *	Negative	Inverse association: higher sweets consumption associated with lower BMI
PBF (%)	+0.032	Positive	Negligible positive, non-significant
VFL	−0.050	Negative	Negligible, non-significant
SMM	−0.120 *	Negative	Negative association—higher sweets consumption associated with lower SMM

Significance: (*) *p* < 0.05; The inverse BMI result is explained by BMI conflating muscle mass with fat mass [5,14].

**Table 5:** Dietary consumption by BMI vs. PBF categories (Mann–Whitney U).

Dietary Item	BMI Normal	BMI High	*p*	PBF Normal	PBF High	*p*
Sweets/Snacks	3.74 ± 1.75	3.46 ± 1.65	0.165	3.64 ± 1.73	3.71 ± 1.72	0.781
White bread	4.11 ± 1.82	4.23 ± 1.66	0.860	4.15 ± 1.78	4.11 ± 1.77	0.814
Dairy	4.56 ± 1.69	4.48 ± 1.60	0.494	4.46 ± 1.74	4.73 ± 1.42	0.516
Fish	2.13 ± 1.58	1.87 ± 1.55	0.230	2.04 ± 1.54	2.11 ± 1.65	0.811
Poultry	4.40 ± 1.58	4.66 ± 1.57	0.132	4.37 ± 1.61	4.75 ± 1.45	0.058
Cheese	4.18 ± 1.63	4.13 ± 1.58	0.655	4.08 ± 1.63	4.39 ± 1.56	0.134
Eggs	4.10 ± 1.62	4.14 ± 1.42	0.839	4.13 ± 1.57	4.06 ± 1.56	0.681
Fruits/Veg	4.29 ± 1.67	4.19 ± 1.57	0.456	4.24 ± 1.67	4.33 ± 1.57	0.780

Cohen’s d effect sizes ranged from 0.02 to 0.16 for BMI comparisons and 0.01 to 0.12 for PBF comparisons. No significant group differences were observed for any food group under either classification (all *p* > 0.05).

**Table 6:** Cross-tabulation of BMI vs. PBF categories (N = 285). Chi-square = 40.79, *p* < 0.001; Cohen’s κ = 0.39 (fair agreement).

	PBF Normal	PBF High	Total
BMI Normal	171 (60.0%)	35 (12.3%)	206
BMI High	35 (12.3%)	44 (15.4%)	79
Total	206	79	285

Agreement: 75.4%. Highlighted: BMI-Normal but PBF-High individuals (false negatives by BMI).

**Table 7:** Multiple regression analysis: predictors of body composition.

Outcome/Predictor	β	SE	t	*p*-Value	VIF
BMI (R^2^ = 0.096, Adj. R^2^ = 0.090)					
Age	0.406	0.13	3.09	0.002 **	1.11
Sex (Female = 0, Male = 1)	1.891	0.44	4.26	<0.001 ***	1.07
Body Fat % (R^2^ = 0.343, Adj. R^2^ = 0.336)					
Age	0.826	0.29	2.86	0.005 **	1.11
Sex (Female = 0, Male = 1)	−11.411	0.97	−11.71	<0.001 ***	1.07
Fish Consumption	−0.424	0.31	−1.38	0.169	1.23
Visceral Fat Level (R^2^ = 0.068, Adj. R^2^ = 0.061)					
Age	0.448	0.16	2.83	0.005 **	1.11
Sex (Female = 0, Male = 1)	−1.931	0.53	−3.62	<0.001 ***	1.07
PBF with MET (R^2^ = 0.347)					
Age	0.770	0.2913	2.64	0.009 **	1.11
Sex (Female = 0, Male = 1)	−11.199	0.9856	−11.36	<0.001 ***	1.07
Fish Consumption	−0.406	0.3072	−1.32	0.188	1.23
MET (per min/week)	−0.0003	0.0002	−1.39	0.165	—

β, unstandardized coefficient; SE, standard error. N = 285 (complete cases). Significance: (**) *p* < 0.01; and (***) *p* < 0.001. The final panel includes MET as a covariate; MET did not reach statistical significance (*p* = 0.165).

**Table 8:** Sex differences in selected variables.

Variable	Males (*n* = 121)	Females (*n* = 164)	*p*-Value
Raw Fruits/Vegetables (0–6)	3.95 (SD 1.58)	4.49 (SD 1.65)	0.0010 **
Sweets/Snacks (0–6)	3.49 (SD 1.76)	3.79 (SD 1.69)	0.1529
Weight (kg)	80.8 (SD 13.8)	62.7 (SD 11.4)	<0.001 ***
Skeletal Muscle Mass (kg)	37.1 (SD 5.6)	23.9 (SD 4.1)	<0.001 ***
Body Fat (%)	18.4 (SD 8.0)	29.6 (SD 8.1)	<0.001 ***

Values are means (SD). Significance: (**) *p* < 0.01; (***) *p* < 0.001 by Mann–Whitney U test.

**Table 9:** Spearman’s rank correlation: MET-min/week vs. body composition.

Variable	rho	*p*-Value	Interpretation
Percent of Body Fat (PBF)	−0.177	0.003 **	More activity = Lower body fat
Skeletal Muscle Mass (SMM)	+0.186	0.002 **	More activity = Higher muscle mass
Fat-Free Mass (FFM)	+0.186	0.002 **	More activity = Higher lean mass
Body Fat Mass (BFM)	−0.120	0.042 *	More activity = Lower fat mass
BMI	+0.060	0.310	Not significant

Significance: (*) *p* < 0.05; (**) *p* < 0.01. Key finding: MET correlates with true adiposity (PBF) but not with BMI.

**Table 10:** Binary logistic regression model for predictors of normal BMI with elevated body fat (normal BMI group, *n* = 206).

Variable	B	SE	Wald	*df*	*p*-Value	OR	95% CI
Intercept	−4.142	2.35	3.10	1	0.078	0.02	—
Age	0.151	0.11	1.88	1	0.170	1.16	0.94–1.44
Sex (Female)	2.464	0.75	10.85	1	0.001 *	11.75	2.71–50.90
Physical Activity (MET)	−0.000	0.00	0.15	1	0.699	1.00	1.00–1.00
Sweets Frequency	0.063	0.12	0.28	1	0.598	1.07	0.84–1.35

Model χ^2^ = 22.52, *df* = 4, and *p* < 0.001; Nagelkerke R^2^ = 0.173. B = unstandardized coefficient; SE = standard error; OR = odds ratio; and CI = confidence interval. (*) *p* < 0.05.

## Data Availability

The data presented in this study are available on request from the corresponding author. The data are not publicly available due to privacy restrictions in accordance with GDPR.

## Abbreviations

The following abbreviations are used in this manuscript:

BIA	Bioelectrical Impedance Analysis
BFM	Body Fat Mass
BMI	Body Mass Index
BMR	Basal Metabolic Rate
FFM	Fat-Free Mass
FFQ	Food Frequency Questionnaire
GDPR	General Data Protection Regulation
GYEMSZI	Hungarian National Institute for Quality and Organizational Development in Healthcare and Medicines
IPAQ-SF	International Physical Activity Questionnaire—Short Form
IQR	Interquartile Range
MET	Metabolic Equivalent
PBF	Percent of Body Fat
SMM	Skeletal Muscle Mass
TBW	Total Body Water
SD	Standard Deviation
VFL	Visceral Fat Level
WHO	World Health Organization
WHR	Waist-Hip Ratio
